# The association between atherogenic index of plasma and all-cause mortality and cardiovascular disease-specific mortality in hypertension patients: a retrospective cohort study of NHANES

**DOI:** 10.1186/s12872-023-03451-0

**Published:** 2023-09-11

**Authors:** Gulinuer duiyimuhan, Nuerguli Maimaiti

**Affiliations:** Hypertension Center of People’s Hospital of Xinjiang Uygur Autonomous Region, No.91 Tianchi Road, Tianshan District, 830001 Urumqi, P.R. China

**Keywords:** AIP, All-cause mortality, CVD-specific mortality, NHANES

## Abstract

**Background:**

Atherogenic index of plasma (AIP), a marker of atherosclerosis and cardiovascular disease (CVD), was related to the all-cause mortality and CVD-specific mortality in a U-shape in general population respectively. However, no studies have investigated these associations in hypertensive populations. Herein, this study aims to explore the relationship of AIP and all-cause mortality and CVD-specific mortality in patients with hypertension in order to provide some reference for the risk hierarchical management of hypertension.

**Methods:**

Demographic and clinical data of 17,382 adult patients with hypertension were extracted from the National Health and Nutrition Examination Survey (NHANES) database in 2005–2018 in this retrospective cohort study. We used weighted univariate COX regression analysis to screen the covariates, and that weighted univariate and multivariate COX regression analyses to explore the association between AIP and all-cause mortality and CVD-specific mortality with hazard ratios (HRs) and 95% confidence intervals (CIs). Subgroup analyses of age, gender, body mass index (BMI), CVD, diabetes mellitus (DM), antihyperlipidemic agents use, and hypotensive drugs use were also performed for further exploration of these relationships.

**Results:**

The average follow-up time was 97.10 months. A total of 2,844 patients died, and 971 of them died due to CVD. After adjusting for age, race, education level, marital status, poverty-income ratio (PIR), smoking, BMI, physical activity, antihyperlipidemic agents, DM, CVD, hypotensive drugs, estimated glomerular filtration rate (eGFR), and total energy intake, we found that both low [HR = 1.18, 95%CI: (1.07–1.32)] and high [HR = 1.17, 95%CI: (1.03–1.33)] levels of AIP were linked to an increased risk of all-cause mortality, and the U-shape association between AIP and CVD-specific mortality was also found [low AIP level: HR = 1.26, 95%CI: (1.05–1.51); high AIP level: HR = 1.26, 95%CI: (1.06–1.49)]. Furthermore, these relationships were existed in patients whose BMI > 25, were non-Hispanic White, with non-CVD, non-DM, non-antihyperlipidemic agents, and used hypertension drug (all *P* < 0.05).

**Conclusion:**

AIP was associated with both all-cause mortality and CVD-specific mortality in patients with hypertension, but the specific role of AIP in prognosis in hypertensive populations is needed further exploration.

## Background

Hypertension is a chronic disease with high prevalence and is the leading preventable risk factor for cardiovascular diseases (CVD) [1, 2]. Globally, more than one billion people are considered hypertensive and the prevalence is expected to reach 29.2% by 2025 [3, 4]. Comparing to the general population, patients with hypertension have increased risks of all-cause mortality [5]. The prevention and management of hypertension has become a major public health challenge.

In addition to hypertension, dyslipidemia is also a risk factor for CVD, and is associated with the risk of hypertension occurrence [4]. Atherogenic index of plasma (AIP), namely total cholesterol (TC) and high-density lipoprotein cholesterol (HDL-C) ratio, is a common indicator of lipid metabolism and a marker of atherosclerosis and CVD [6]. A 10-year prospective cohort study by Dong et al. [7] on the relationships of lipid profiles with CVD and mortality found a U-shape relationship between AIP and the CVD-specific mortality in a community-dwelling population in China. Another study explored the effect of AIP on mortality risk in the general population, the restricted cubic spline (RCS) analysis showed that a U-shape association between AIP and all-cause mortality but the non-linear association between AIP and CVD-specific mortality was not significant [8].

AIP is a novel predictive biomarker for cardiovascular illnesses, and is also a robust biomarker of dyslipidemia and atherosclerosis, has been used to quantify comprehensive lipid levels [9]. Studies have indicated it could be recommended as a potential biomarker in the early diagnosis of CVD events [9, 10]. Nevertheless, no studies have investigated the association of AIP with all-cause and CVD-specific mortality in hypertensive populations. Considering the role of lipid metabolism in hypertension and CVD and the poor prognosis of hypertension, a mature and convenient biomarker for the prognosis of patients with hypertension is in need clinically.

Herein, this study aims to explore the relationship between AIP and all-cause mortality and CVD-specific mortality in patients with hypertension, and further assess these relationships in subgroups of age, gender, body mass index (BMI), and complications. We hope this study can help to provide some references for the risk hierarchical management in hypertensive populations.

## Methods

### Study design and population

Data in this retrospective cohort study were extracted from the National Health and Nutrition Examination Survey (NHANES) database in 2005–2018. NHANES is a multipurpose research program done by the National Center for Health Statistics (NCHS) that assesses the health and nutritional status of adults and children in the United States [11]. The survey regularly collects data of approximately 5,000 persons from 15 areas since 1999 (datasets of every two years were incorporated into a cycle) that includes a household interview followed by a standardized physical examination in a mobile examination center (MEC). A stratified multistage sampling design with a weighting scheme based on the selection of counties, blocks, households, and persons within households is used by NHANES to represent the civilian, non-institutionalized population in the United States and accurately estimate disease prevalence. Details of study implementation are available for online access: https://wwwn.cdc.gov/nchs/nhanes/tutorials/module2.aspx.

A total of 22,628 patients with hypertension were initially included. The exclusion criteria were (1) aged < 18 years old, and (2) missing information of serum TC or HDL or mortality. We also excluded individuals who missing information of study variables including education level, marital status, physical activity, Dietary Approaches to Stop Hypertension (DASH) score, smoking, BMI, and CVD. After excluding patients who lost to the follow-up, finally, 17,382 of them were eligible.

All methods for this study were performed in accordance with the relevant guidelines and regulations. Written informed consent was obtained from participants by NHANES. Since NHANES is publicly available, and the data of patients were de-identified, the requirement of ethical approval for this study was waived by the Institutional Review Board of People’s Hospital of Xinjiang Uygur Autonomous Region.

### Definition of atherogenic index of plasma

Blood sample collection and measurement in NHAENS were conducted according to a standardized protocol from the Centers for Disease Control and Prevention (CDC). Serum HDL-C was measured by direct immunoassay or precipitation [12]. Also, serum TC and HDL-C levels were measured enzymatically with a Hitachi 704 Analyzer (Boehringer Mannheim Diagnostics, Indianapolis, IN, USA) [13]. The AIP was calculated by the serum TC (mmol/L) and HDL-C (mmol/L) ratio. More information of laboratory examination was provided on the website of CDC. We divided the AIP into three levels according to the tertiles in this study, including low level: AIP < 3.25, median level: 3.25 ≤ AIP ≤ 4.34, and high level: AIP > 4.34.

### Variables collection

We extracted variables including age, gender, race, marital status, education level, poverty income ratio (PIR), smoking and drinking status, physical activity, BMI, diabetes mellitus (DM), CVD, antihyperlipidemic agents, hypotensive drugs, estimated glomerular filtration rate (eGFR), DASH score, total energy intake, and survival time from the NHANES database.

Physical activity was translated into energy expenditure. Metabolic equivalent (MET) was calculated based on the questionnaire of physical activity reports (PAQ) in NHANES using the following formula: Energy expenditure (MET·min) = recommended MET × exercise time of corresponding activity (min). Complications including DM, CVD, and dyslipidemia of individuals were diagnosed according to laboratory examination, self-reports and the medication history. DM was defined as fasting blood glucose ≥ 7.0 mmol/L or Glycosylated Hemoglobin (HbAlc) ≥ 6.5% or self-reported DM or receiving hypoglycemic therapy. CVD diagnosis was according to a positive answer to the NHANES multiple choice question (MCQ): “Have you ever been told you had (congestive) heart failure, coronary heart disease, angina/angina pectoris, heart attack and stroke " or using of cardiovascular drugs. The information of antihyperlipidemic agents was from self-reported, or the records of prescription medications-drug information (serial number 358 − 19). The eGFR was calculated using the following formula: estimated GFR = 175 × standardized serum creatinine (Scr) − 1.154 × age − 0.203 × 1.212 (if black) × 0.742 (if female), where GFR is expressed as mL/min/1.73 m^2^ of body surface area and Scr is expressed in mg/dL [14]. The DASH dietary score was calculated based on the nine-item, nutrient-based DASH index: protein, fiber, magnesium, calcium, potassium, total fat, saturated fat, cholesterol, and sodium. The optimal micronutrient targets are energy adjusted per 1000 kcal. Meeting the goal for each component provides one point, meeting an intermediate goal between the DASH diet goal and the nutrient content of the DASH control diet provides 0.5 points, and meeting neither goal gives zero points. In addition, this pattern score ranges between 0 and 9 points, and higher scores indicates greater adherence to the DASH dietary pattern [15]. The total energy intake was calculated according to the two 24-hour dietary recalls in the NHANES. The first 24-hour recall interview was conducted in person in the mobile exam centers (MEC) by trained interviewers, and the second interview was performed by telephone or mail three to ten days later. We used the records of first 24-hour recall in this study.

### Outcome and follow-up

The study outcomes were all-cause mortality and CVD-specific mortality. The information of mortality in the NHANES in 2005–2018 were linked to mortality data from the National Death Index death certificate records (www.cdc.gov/nchs/data-linkage/mortality-public.htm) until December 31, 2019, matched using a probabilistic matching algorithm to identify mortality status [16]. CVD-specific mortality (codes I00–I09, I11, I13, I20–I51, and I60–I69) was estimated using the International Classification of Disease Tenth Revision (ICD-10). The follow-up ended if the patients died or until the 31st December 2019.

### Statistical analysis

Continuous data were described by mean ± standard error (mean ± SE), and weighted t test was used for comparation between groups. Enumeration data were expressed as number with constituent ratio [N (%)], and chi-square test for the comparison. Since we merged seven cycles of NHANES datasets, and to enable more excellent statistical reliability, the MEC sample weights (WTMEC2YR/7) were used for weighted analyses (https://wwwn.cdc.gov/nchs/nhanes/tutorials/default.aspx).

Weighted univariate COX regression analyses were used to screen the covariates that related to all-cause and CVD-specific mortality respectively. Weighted univariate and multivariate COX regression analyses were used to explore the association between AIP and all-cause and CVD-specific mortality. Restricted cubic spline (RCS) curves were used to show the relationships between AIP and the two outcomes. Model 1 adjusted for age (years) and race. Model 2 adjusted for age, race, education level, marital status, PIR, smoking, BMI (kg/m^2^), physical activity, antihyperlipidemic agents, DM, CVD, hypotensive drugs, eGFR [mL/ (min·1.73 m^2^)], and energy intake (kcal). We also explored these relationships in age, gender, BMI, CVD, DM, antihyperlipidemic agents use, and hypotensive drugs use subgroups.

The evaluation indexes were hazard ratios (HRs) and 95% confidence intervals (CIs). Two-sided *P* < 0.05 was considered significant. Statistical analysis was performed using SAS 9.4 (SAS Institute, Cary, NC, USA) and R version 4.2.0 (Institute for Statistics and Mathematics, Vienna, Austria). Missing data were deleted.

## Results

### Characteristics of hypertension patients

**Fig. 1:**
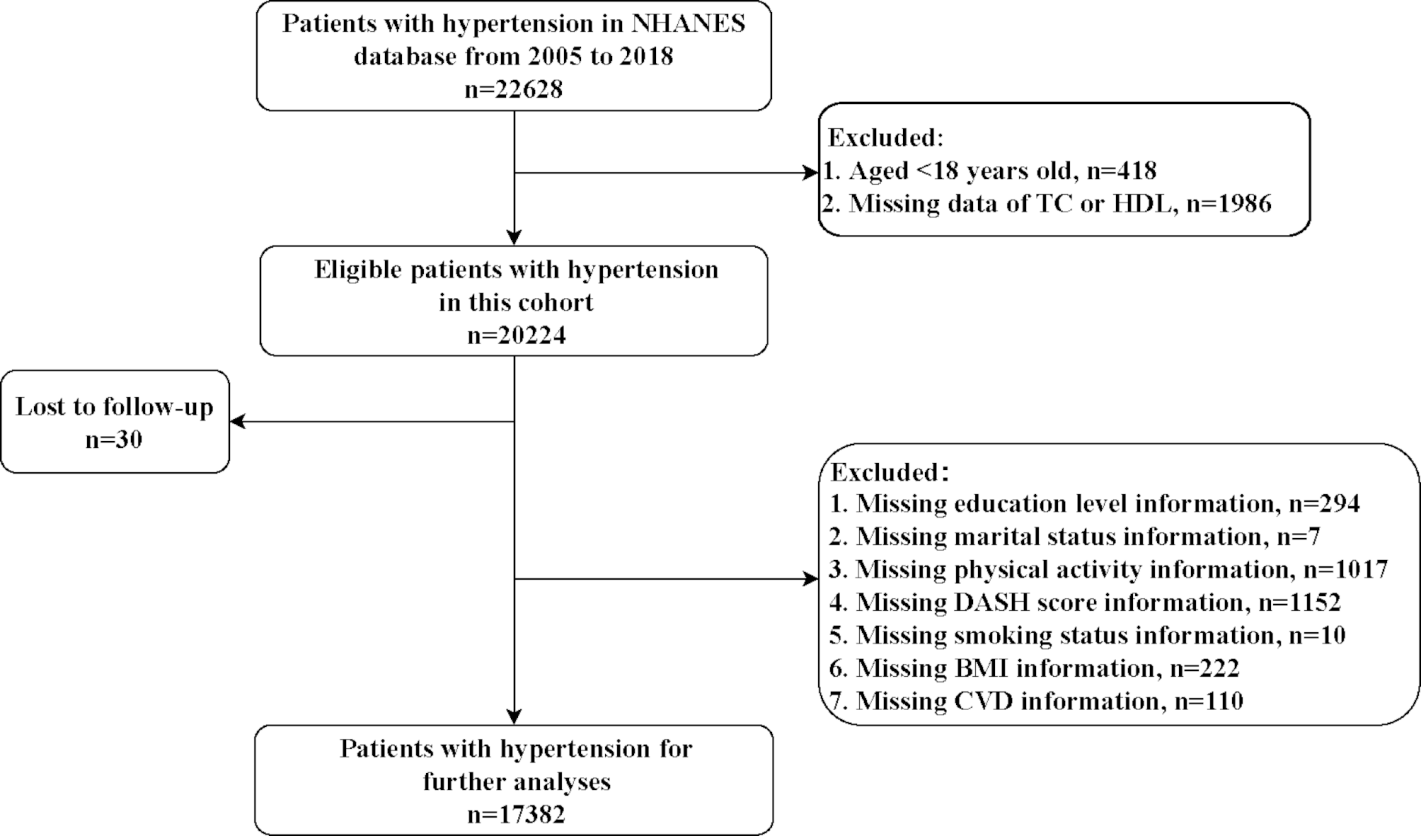
Flow chart of the participants screening

Figure 1 is the flow chart of the participants screening. A total of 22,628 patients with hypertension were initially included. Then we excluded those who aged < 18 years old (n = 418), and without information of serum TC or HDL (n = 1986). In the eligible patients, 30 of them lost to the follow-up. Those who missing information of education level (n = 294), marital status (n = 7), physical activity (n = 1017), DASH score (n = 1152), smoking status (n = 10), BMI (n = 222), and CVD (n = 110) were also excluded. Finally, 17,382 of hypertension patients were included for further analyses.

Table 1 shows the characteristics of patients with hypertension. The average follow-up time was approximately 97 months. A total of 2,844 patients died, and 971 of them died due to CVD. Among the patients, 9,050 (52.05%) were males and 8,332 (47.95%) were females. Most of them were non-Hispanic White (70.55%), BMI > 25 (79.98%), and not have CVD (85.37%). Additionally, age, marital status, education level, PIR, antihyperlipidemic agents use, hypotensive drugs use, eGFR, DASH score, and total energy intake were all different among the three AIP level groups (all *P* < 0.05).

**Table 1:** Characteristics of patients with hypertension

Variables	Total (n = 17,382)	AIP levels			*P*
		< 3.25 (n = 5736)	3.25–4.34 (n = 5732)	> 4.34 (n = 5914)	
Age, years, n (%)					< 0.001
<40	2798 (18.54)	769 (15.59)	841 (17.00)	1188 (22.73)	
40–60	5937 (40.83)	1601 (33.86)	1934 (41.21)	2402 (46.90)	
≥60	8647 (40.63)	3366 (50.55)	2957 (41.79)	2324 (30.37)	
Gender, n (%)					< 0.001
Male	9050 (52.05)	2449 (40.79)	2887 (49.63)	3714 (64.74)	
Female	8332 (47.95)	3287 (59.21)	2845 (50.37)	2200 (35.26)	
Race, n (%)					< 0.001
Mexican American	2250 (6.34)	551 (4.41)	766 (6.35)	933 (8.12)	
Other Hispanic	1568 (4.52)	404 (3.28)	525 (4.40)	639 (5.79)	
Non-Hispanic White	7801 (70.55)	2526 (70.60)	2542 (70.77)	2733 (70.30)	
Non-Hispanic Black	4153 (12.04)	1724 (15.31)	1381 (12.02)	1048 (9.04)	
Other race-including multi-racial	1610 (6.54)	531 (6.40)	518 (6.46)	561 (6.75)	
Marital status, n (%)					< 0.001
Married	9380 (58.82)	2841 (55.56)	3199 (60.60)	3340 (60.13)	
Widowed	2015 (8.86)	874 (12.04)	648 (8.91)	493 (5.87)	
Divorced	2259 (11.98)	751 (12.04)	729 (11.42)	779 (12.47)	
Separated	610 (2.50)	217 (2.69)	200 (2.76)	193 (2.08)	
Never married	2076 (11.64)	712 (11.69)	651 (11.00)	713 (12.20)	
Living with partner	1042 (6.20)	341 (5.98)	305 (5.31)	396 (7.24)	
Education level, n (%)					< 0.001
Less Than 9th Grade	1975 (5.66)	576 (5.05)	677 (5.52)	722 (6.37)	
9-11th Grade	2464 (10.55)	791 (10.01)	810 (10.64)	863 (10.97)	
High School Grad/GED or Equivalent	4197 (24.61)	1367 (23.33)	1363 (24.58)	1467 (25.81)	
Some College or AA degree	5134 (32.33)	1668 (31.10)	1712 (33.00)	1754 (32.82)	
College Graduate or above	3612 (26.85)	1334 (30.52)	1170 (26.27)	1108 (24.03)	
PIR, n (%)					0.002
≤1	2989 (11.10)	919 (10.33)	976 (10.56)	1094 (12.32)	
>1	12,806 (81.48)	4267 (81.33)	4245 (82.19)	4294 (80.95)	
Unknown	1587 (7.42)	550 (8.35)	511 (7.25)	526 (6.73)	
Smoking, n (%)					0.230
Yes	8365 (48.20)	2696 (47.66)	2729 (47.57)	2940 (49.30)	
No	9017 (51.80)	3040 (52.34)	3003 (52.43)	2974 (50.70)	
Drinking, n (%)					0.654
Yes	16,417 (95.42)	5393 (95.15)	5419 (95.54)	5605 (95.55)	
No	965 (4.58)	343 (4.85)	313 (4.46)	309 (4.45)	
Physical activity, MET*min/week, n (%)					0.761
≤750	7190 (36.99)	2365 (36.61)	2407 (36.84)	2418 (37.47)	
>750	10,192 (63.01)	3371 (63.39)	3325 (63.16)	3496 (62.53)	
BMI, kg/m^2^, n (%)					< 0.001
≤25	3588 (20.02)	1896 (34.17)	1037 (17.61)	655 (9.27)	
>25	13,794 (79.98)	3840 (65.83)	4695 (82.39)	5259 (90.73)	
DM, n (%)					0.064
No	12,746 (78.72)	4266 (80.15)	4173 (78.38)	4307 (77.74)	
Yes	4636 (21.28)	1470 (19.85)	1559 (21.62)	1607 (22.26)	
CVD, n (%)					< 0.001
No	14,355 (85.37)	4579 (82.76)	4736 (85.75)	5040 (87.40)	
Yes	3027 (14.63)	1157 (17.24)	996 (14.25)	874 (12.60)	
Antihyperlipidemic agents, n (%)					< 0.001
No	11,310 (67.79)	3247 (59.38)	3670 (66.51)	4393 (76.77)	
Yes	6072 (32.21)	2489 (40.62)	2062 (33.49)	1521 (23.23)	
Hypotensive drugs, n (%)					< 0.001
No	6300 (39.88)	1801 (35.27)	1974 (38.15)	2525 (45.78)	
Yes	11,082 (60.12)	3935 (64.73)	3758 (61.85)	3389 (54.22)	
eGFR, mL/ (min·1.73 m^2^), Mean (S.E)	92.19 (0.32)	90.94 (0.48)	92.15 (0.42)	93.38 (0.42)	< 0.001
DASH score, Mean (S.E)	2.18 (0.02)	2.31 (0.03)	2.15 (0.03)	2.09 (0.03)	< 0.001
Energy intake, kcal, Mean (S.E)	2073.96 (9.50)	1972.78 (12.82)	2052.93 (14.51)	2187.30 (17.84)	< 0.001
Survival time, months, Mean (S.E)	97.10 (1.57)	91.93 (1.91)	96.70 (2.17)	102.27 (1.97)	< 0.001
Mortality, n (%)					< 0.001
Survival	14,538 (86.36)	4666 (84.12)	4857 (87.96)	5015 (86.90)	
CVD-specific mortality	971 (4.65)	364 (5.51)	294 (3.94)	313 (4.54)	
Other-cause mortality	1873 (8.99)	706 (10.37)	581 (8.10)	586 (8.56)	

AIP: atherogenic index of plasma, PIR: poverty income ratio, MET: metabolic equivalent, BMI: body mass index, DM: diabetes mellitus, CVD: cardiovascular disease, eGFR: estimated glomerular filtration rate, DASH: Dietary Approaches to Stop Hypertension, S.E: standard error

### Association between AIP and all-cause and CVD-specific mortality

Table 2 shows the covariates that associated with all-cause mortality and CVD-specific mortality respectively. Age, race, education level, marital status, PIR, smoking, BMI, physical activity, antihyperlipidemic agents, DM, CVD, hypotensive drugs, eGFR, and total energy intake were all significantly associated with both all-cause mortality and CVD-specific mortality, and they were further included in the adjusted model.

**Table 2:** Screening of the covariates related to all-cause and CVD-specific mortality

Variables	All-cause mortality		CVD-specific mortality	
	HR (95% CI)	*P*	HR (95% CI)	*P*
Age				
<40	Ref		Ref	
40–60	1.52 (1.23–1.87)	< 0.001	1.44 (1.03–2.01)	0.032
≥60	4.22 (3.52–5.07)	< 0.001	4.32 (3.14–5.95)	< 0.001
Gender				
Male	Ref		Ref	
Female	0.97 (0.88–1.07)	0.582	0.90 (0.76–1.07)	0.242
Race				
Mexican American	Ref		Ref	
Other Hispanic	1.01 (0.78–1.30)	0.945	1.17 (0.79–1.72)	0.435
Non-Hispanic White	1.61 (1.37–1.90)	< 0.001	1.57 (1.21–2.03)	< 0.001
Non-Hispanic Black	1.36 (1.14–1.61)	< 0.001	1.39 (1.05–1.84)	0.023
Other race - including multi-racial	1.13 (0.89–1.43)	0.314	1.39 (0.97–1.99)	0.074
Education level				
Less Than 9th Grade	Ref		Ref	
9-11th Grade	0.72 (0.61–0.86)	< 0.001	0.59 (0.45–0.78)	< 0.001
High School Grad/GED or Equivalent	0.68 (0.58–0.79)	< 0.001	0.67 (0.51–0.87)	0.003
Some College or AA degree	0.53 (0.44–0.63)	< 0.001	0.52 (0.40–0.67)	< 0.001
College Graduate or above	0.44 (0.36–0.54)	< 0.001	0.46 (0.34–0.61)	< 0.001
Marital status				
Married	Ref		Ref	
Widowed	3.08 (2.76–3.43)	< 0.001	3.19 (2.60–3.91)	< 0.001
Divorced	1.23 (1.05–1.45)	0.012	1.09 (0.83–1.43)	0.549
Separated	1.14 (0.90–1.46)	0.284	1.37 (0.90–2.09)	0.143
Never married	0.78 (0.64–0.95)	0.015	0.79 (0.61–1.04)	0.095
Living with partner	0.75 (0.58–0.96)	0.022	0.44 (0.27–0.71)	< 0.001
PIR				
≤1	Ref		Ref	
>1	0.76 (0.66–0.88)	< 0.001	0.81 (0.64–1.04)	0.093
Unknown	0.78 (0.65–0.95)	0.011	0.55 (0.38–0.79)	0.001
Smoking				
Yes	Ref		Ref	
No	0.68 (0.62–0.75)	< 0.001	0.82 (0.70–0.95)	0.008
Drinking				
Yes	Ref		Ref	
No	1.01 (0.80–1.27)	0.958	0.76 (0.48–1.22)	0.255
BMI				
≤25	Ref		Ref	
>25	0.74 (0.66–0.83)	< 0.001	0.78 (0.66–0.92)	0.003
Physical activity				
≤750	Ref		Ref	
>750	0.55 (0.50–0.61)	< 0.001	0.54 (0.45–0.65)	< 0.001
Antihyperlipidemic agents				
No	Ref		Ref	
Yes	1.77 (1.63–1.92)	< 0.001	1.99 (1.72–2.31)	< 0.001
DM				
No	Ref		Ref	
Yes	1.86 (1.70–2.05)	< 0.001	1.90 (1.61–2.23)	< 0.001
CVD				
No	Ref		Ref	
Yes	2.81 (2.52–3.14)	< 0.001	3.27 (2.68–3.98)	< 0.001
Hypertension treatment				
No	Ref		Ref	
Yes	2.04 (1.82–2.28)	< 0.001	2.25 (1.86–2.73)	< 0.001
eGFR	0.56 (0.53–0.58)	< 0.001	0.53 (0.48–0.58)	< 0.001
DASH score	1.01 (0.97–1.06)	0.643	1.05 (0.97–1.14)	0.267
Energy intake	0.84 (0.79–0.89)	< 0.001	0.84 (0.76–0.92)	< 0.001

CVD: cardiovascular disease, HR: hazard ratio, CI: confidence interval, Ref: reference, PIR: poverty income ratio, BMI: body mass index, DM: diabetes mellitus, eGFR: estimated glomerular filtration rate, DASH: Dietary Approaches to Stop Hypertension

**Fig. 2:**
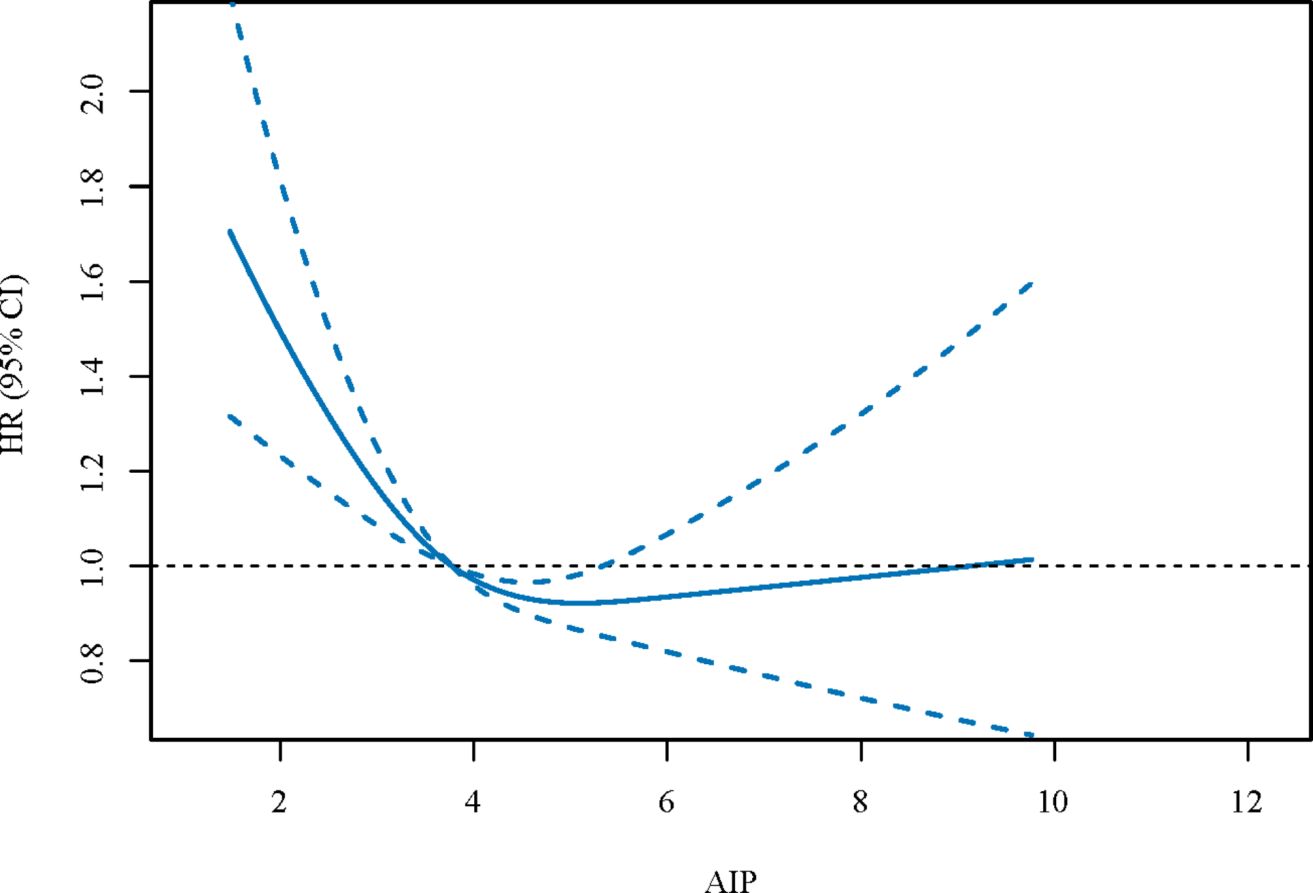
The RCS curves of AIP and all-cause mortality

**Fig. 3:**
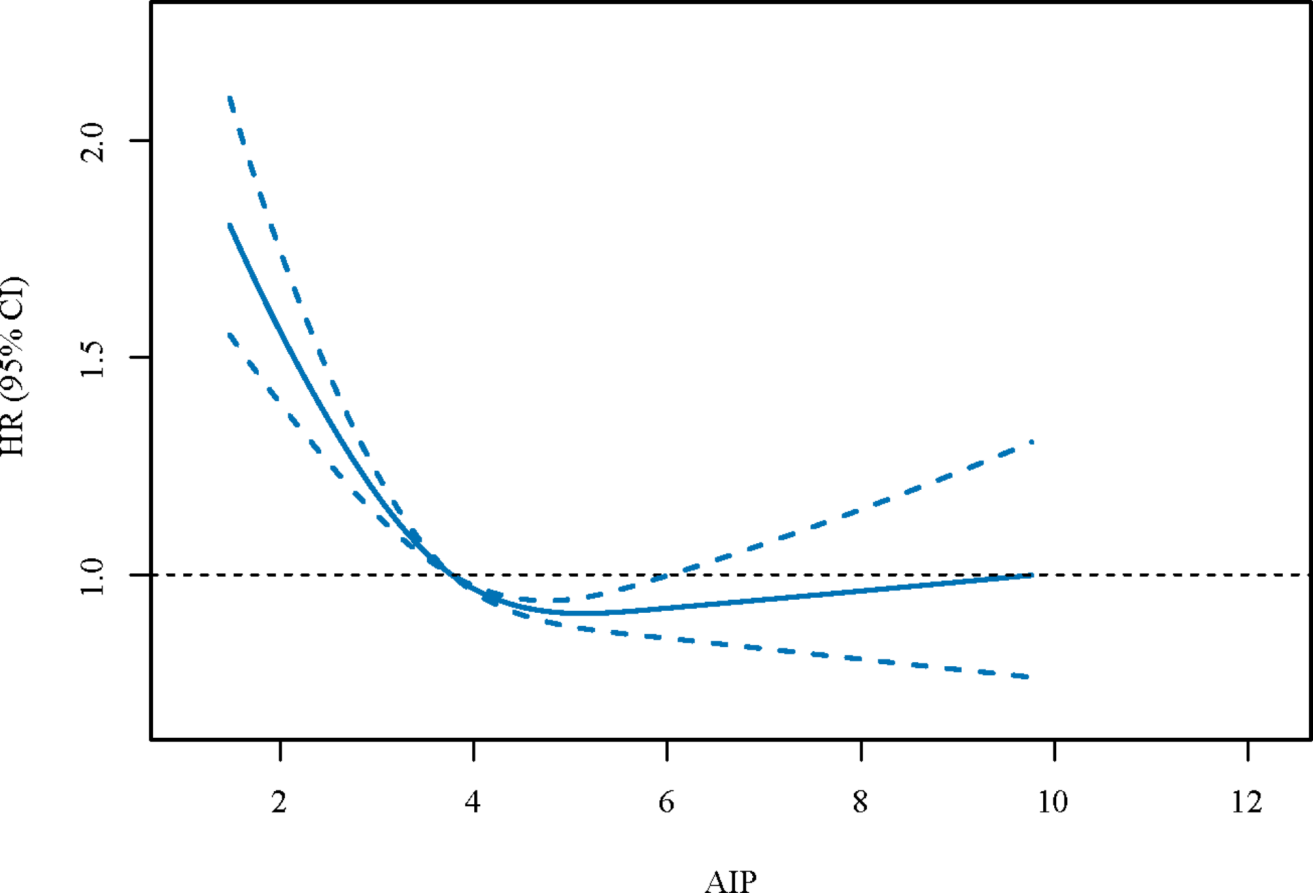
The RCS curves of AIP and CVD-specific mortality

We then explored the relationships between AIP and all-cause mortality, and between AIP and CVD-specific mortality (Table 3). After adjusting for the covariables, we found that both low [HR = 1.18, 95%CI: (1.07–1.32)] and high [HR = 1.17, 95%CI: (1.03–1.33)] levels of AIP were linked to an increased risk of all-cause mortality. Similarly, the U-shape association between AIP and CVD-specific mortality was also found [low AIP level: HR = 1.26, 95%CI: (1.05–1.51); high AIP level: HR = 1.26, 95%CI: (1.06–1.49)]. Figures 2 and 3 were the RCS curves of AIP and all-cause mortality, and AIP and CVD-specific mortality respectively.

**Table 3:** Association between AIP and all-cause and CVD-specific mortality

AIP levels	Crude model		Model 1		Model 2	
	h (95% CI)	*P*	HR (95% CI)	*P*	HR (95% CI)	*P*
**All-cause mortality**						
3.25–4.34	Ref		Ref		Ref	
< 3.25	1.40 (1.26–1.56)	< 0.001	1.23 (1.12–1.37)	< 0.001	1.18 (1.07–1.32)	0.002
> 4.34	1.02 (0.89–1.17)	0.764	1.24 (1.08–1.41)	0.002	1.17 (1.03–1.33)	0.016
**CVD-specific mortality**						
3.25–4.34	Ref		Ref		Ref	
< 3.25	1.49 (1.23–1.80)	< 0.001	1.31 (1.09–1.56)	0.004	1.26 (1.05–1.51)	0.015
> 4.34	1.08 (0.90–1.29)	0.403	1.31 (1.10–1.57)	0.003	1.26 (1.06–1.49)	0.009

AIP: atherogenic index of plasma, CVD: cardiovascular disease, HR: hazard ratio, CI: confidence interval, Ref: reference

Model 1: adjusted for age and gender;

Model 2: adjusted for age, race, education level, marital status, PIR, smoking, BMI, physical activity, antihyperlipidemic agents, DM, CVD, hypotensive drugs, eGFR, and energy intake

### Relationships of AIP and all-cause mortality and CVD-specific mortality in different subgroups

We further explored these associations in different subgroups (Table 4). After adjusting for covariates, the U-shape relationships between AIP and all-cause mortality, and between AIP and CVD-specific mortality were also found in patients with hypertension whose BMI > 25, were non-Hispanic White, with non-CVD, non-DM, and non-antihyperlipidemic agents, and used hypertension drug (all *P* < 0.05). Differently, the U-shape association between AIP level and all-cause mortality was found in female patients (HR = 1.16 for low level and HR = 1.20 for high level), while the U-shape association between AIP level and of CVD-specific mortality was found in patients aged ≥ 60 years old (HR = 1.28 for low level and HR = 1.36 for high level).

**Table 4:** Relationships of AIP and all-cause and CVD-specific mortality in age, gender, race, BMI, CVD, DM, antihyperlipidemic agents use and hypotensive drugs use subgroups

Subgroups	AIP levels	All-cause mortality		CVD-specific mortality	
		HR (95% CI)	*P*	HR (95% CI)	*P*
Age < 40	3.25–4.34	Ref		Ref	
	< 3.25	1.55 (0.88–2.73)	0.129	0.98 (0.44–2.20)	0.967
	> 4.34	1.37 (0.85–2.21)	0.190	0.70 (0.33–1.49)	0.355
40 ≤ Age < 60	3.25–4.34	Ref		Ref	
	< 3.25	1.30 (0.98–1.72)	0.068	1.19 (0.74–1.90)	0.472
	> 4.34	1.45 (1.11–1.88)	0.006	1.21 (0.80–1.81)	0.363
Age ≥ 60	3.25–4.34	Ref		Ref	
	< 3.25	1.10 (0.98–1.23)	0.121	1.28 (1.05–1.57)	0.016
	> 4.34	1.05 (0.91–1.21)	0.486	1.36 (1.11–1.68)	0.004
Male	3.25–4.34	Ref		Ref	
	< 3.25	1.27 (1.10–1.48)	0.001	1.37 (1.06–1.77)	0.015
	> 4.34	1.12 (0.94–1.32)	0.196	1.17 (0.90–1.53)	0.232
Female	3.25–4.34	Ref		Ref	
	< 3.25	1.16 (1.00-1.34)	0.044	1.23 (0.95–1.59)	0.112
	> 4.34	1.20 (1.00-1.44)	0.046	1.29 (1.03–1.63)	0.028
BMI ≤ 25	3.25–4.34	Ref		Ref	
	< 3.25	1.05 (0.87–1.27)	0.627	1.03 (0.72–1.47)	0.88
	> 4.34	0.99 (0.76–1.30)	0.962	1.11 (0.73–1.68)	0.633
BMI > 25	3.25–4.34	Ref		Ref	
	< 3.25	1.26 (1.09–1.45)	0.001	1.40 (1.10–1.78)	0.007
	> 4.34	1.22 (1.04–1.43)	0.013	1.31 (1.05–1.63)	0.017
Mexican American	3.25–4.34	Ref		Ref	
	< 3.25	1.39 (0.94–2.05)	0.097	1.16 (0.66–2.03)	0.611
	> 4.34	1.18 (0.84–1.66)	0.345	1.20 (0.69–2.06)	0.516
Other Hispanic	3.25–4.34	Ref		Ref	
	< 3.25	1.02 (0.66–1.56)	0.930	1.01 (0.42–2.45)	0.977
	> 4.34	0.67 (0.42–1.08)	0.100	0.63 (0.30–1.31)	0.214
Non-Hispanic White	3.25–4.34	Ref		Ref	
	< 3.25	1.22 (1.06–1.41)	0.005	1.34 (1.06–1.69)	0.013
	> 4.34	1.18 (1.00-1.39)	0.048	1.28 (1.03–1.60)	0.024
Non-Hispanic Black	3.25–4.34	Ref		Ref	
	< 3.25	1.11 (0.92–1.33)	0.284	1.25 (0.91–1.71)	0.174
	> 4.34	1.38 (1.08–1.77)	0.010	1.36 (0.93-2.00)	0.110
Other race-including multi-racial	3.25–4.34	Ref		Ref	
	< 3.25	0.72 (0.38–1.36)	0.312	0.75 (0.25–2.25)	0.608
	> 4.34	1.01 (0.61–1.67)	0.958	1.36 (0.53–3.45)	0.521
Non-CVD	3.25–4.34	Ref		Ref	
	< 3.25	1.29 (1.14–1.46)	< 0.001	1.33 (1.04–1.70)	0.026
	> 4.34	1.25 (1.07–1.46)	0.005	1.29 (1.03–1.61)	0.026
CVD	3.25–4.34	Ref		Ref	
	< 3.25	0.99 (0.81–1.20)	0.923	1.15 (0.92–1.43)	0.226
	> 4.34	0.94 (0.79–1.13)	0.512	1.10 (0.80–1.50)	0.573
Non-DM	3.25–4.34	Ref		Ref	
	< 3.25	1.26 (1.11–1.44)	< 0.001	1.26 (1.02–1.57)	0.033
	> 4.34	1.19 (1.05–1.35)	0.005	1.27 (1.05–1.55)	0.015
DM	3.25–4.34	Ref		Ref	
	< 3.25	1.01 (0.83–1.22)	0.937	1.24 (0.92–1.67)	0.163
	> 4.34	1.09 (0.82–1.46)	0.534	1.20 (0.84–1.72)	0.319
Non-antihyperlipidemic agents	3.25–4.34	Ref		Ref	
	< 3.25	1.38 (1.19–1.60)	< 0.001	1.37 (1.04–1.81)	0.026
	> 4.34	1.20 (1.00-1.45)	0.049	1.38 (1.12–1.70)	0.003
Antihyperlipidemic agents use	3.25–4.34	Ref		Ref	
	< 3.25	1.01 (0.87–1.19)	0.864	1.13 (0.89–1.45)	0.305
	> 4.34	1.18 (0.97–1.44)	0.095	1.14 (0.83–1.56)	0.430
Non-hypertension drug	3.25–4.34	Ref		Ref	
	< 3.25	1.22 (0.92–1.61)	0.159	1.05 (0.64–1.71)	0.854
	> 4.34	1.10 (0.85–1.42)	0.465	0.88 (0.56–1.39)	0.586
Hypertension drug use	3.25–4.34	Ref		Ref	
	< 3.25	1.18 (1.05–1.32)	0.007	1.34 (1.11–1.61)	0.002
	> 4.34	1.21 (1.04–1.40)	0.012	1.41 (1.17–1.70)	< 0.001

AIP: atherogenic index of plasma, CVD: cardiovascular disease, BMI: body mass index, DM: diabetes mellitus, HR: hazard ratio, CI: confidence interval, Ref: reference

Age subgroup: adjusted for race, education level, marital status, PIR, smoking, BMI, physical activity, antihyperlipidemic agents, DM, CVD, hypotensive drugs, eGFR, and energy intake;

Gender subgroup: adjusted for age, race, education level, marital status, PIR, smoking, BMI, physical activity, antihyperlipidemic agents, DM, CVD, hypotensive drugs, eGFR, and energy intake;

BMI subgroups: adjusted for age, race, education level, marital status, PIR, smoking, physical activity, antihyperlipidemic agents, DM, CVD, hypotensive drugs, eGFR, and energy intake;

Race subgroup: adjusted for age, education level, marital status, PIR, smoking, BMI, physical activity, antihyperlipidemic agents, DM, CVD, hypotensive drugs, eGFR, and energy intake;

CVD subgroup: adjusted for age, race, education level, marital status, PIR, smoking, BMI, physical activity, antihyperlipidemic agents, DM, hypotensive drugs, eGFR, and energy intake;

DM subgroup: adjusted for age, race, education level, marital status, PIR, smoking, BMI, physical activity, antihyperlipidemic agents, CVD, hypotensive drugs, eGFR, and energy intake;

Antihyperlipidemic agents use subgroup: adjusted for age, race, education level, marital status, PIR, smoking, BMI, physical activity, DM, CVD, hypotensive drugs, eGFR, and energy intake;

Hypertension drug use subgroup: adjusted for age, race, education level, marital status, PIR, smoking, BMI, physical activity, antihyperlipidemic agents, DM, CVD, eGFR, and energy intake

## Discussion

In this study, we explored the association between AIP and all-cause mortality and CVD-specific mortality in patients with hypertension. The results showed that after adjusting for the covariates, AIP level was related to both all-cause mortality and CVD-specific mortality in a U-shape. These relationships were also found in patients whose BMI > 25, were non-Hispanic White, with non-CVD, non-DM, and non-antihyperlipidemic agents, and used hypertension drug.

To our knowledge, no study has explored the association between AIP and all-cause mortality and CVD-specific mortality in hypertensive population. Results of previous relevant study remained inconsistent. Dong et al. [7] performed a 10-year prospective cohort study on the associations between lipid profiles and CVD and mortality in a Chinese community-dwelling population, which found a U-shape relationship between AIP and the CVD-specific mortality. Zhou et al. [8] searched in health adults from the 1999–2014 NHANES with the aim of exploring the effect of AIP levels (< 2.86, 2.86–3.46, 3.46–4.12, 4.12–5.07, and > 5.07) on the risk of all-cause mortality and CVD-specific mortality using RCS analyses. Their results showed a U-shape association between AIP and all-cause mortality, but this association between AIP and CVD-specific mortality was not significant. In this study, we found both low and high levels of AIP were linked to increased risks of all-cause and CVD mortality (a U-shape relationship) in patients with hypertension. The AIP has been reported to be an independent risk factor for CVD occurrence and mortality [17]. The potential mechanism of relationship between AIP and CVD mortality could be explained by the association of the index with lipoprotein particle size: it is inversely related to LDL cholesterol particle diameter [18]. Results of some studies showed that small dense LDL cholesterol particles were related to higher risk of CVD [19, 20]. Besides, chronic inflammation leads to lipid metabolism derangement, which may also play an important role in the association between AIP and CVD-specific mortality. The specific mechanism of the effect of low or high AIP level on all-cause mortality and CVD-specific mortality in patients with hypertension is needed to be further explored.

Subgroup analyses of age, gender, race, BMI, DM, CVD, antihyperlipidemic agents use and hypotensive drugs use were also performed for further exploration of this U-shape relationship in different populations. In a study on the relationship between AIP and obesity among adults in Taiwan showed that subjects with high AIP levels exhibited significant differences in systolic blood pressure (SBP), diastolic blood pressure (DBP), waist circumference, fasting plasma glucose, serum HDL-C, serum low-density lipoprotein cholesterol (LDL-C), serum TC and prevalence of DM, hypertension, hyperlipidemia and metabolic syndrome [21]. However, the study in Taiwan was not examine the association of AIP with other parameters in obese and non-obese individuals or in different degrees of obesity. Herein, our study was partly extend exploration of the study in Taiwan, because we found that in obese patients instead of those without obesity, AIP levels were related to both all-cause mortality and CVD-specific mortality. Ethnic difference in serum lipid profile, and may further influence the obesity and even death [22]. We found U-shape relationships between AIP and all-cause mortality, and between AIP and CVD-specific mortality in hypertensive patients who were non-Hispanic White. Obesity has been reported to be a stronger risk factor for all-cause mortality among non-Hispanic Caucasians than for African-Americans, and however, the association between obesity and risks of CHD mortality in different race have not been explored exclusively [23]. Fu et al. [24] confirmed that AIP could be used as an independent predictor for the prognosis of typer 2 diabetes mellitus (T2DM) patients in the long-term follow-up, because comparing with patients without T2DM, patients with T2DM tend to have more cardiovascular risk factors, including hyperlipidemia. In the current study, in hypertensive patients without DM, AIP levels were related to both all-cause mortality and CVD-specific mortality. In addition, we found these associations in patients without CVD, not take antihyperlipidemic agents and used hypertension drug. Actually, hyperglycemia, insulin resistance and hyperinsulinemia can all cause lipid metabolism disorders, oxidative stress and vascular endothelial damage, ultimately leading to the aggravation of coronary atherosclerosis [25]. Due to the AIP is calculated by serum TC and HDL-C ratio, the antihyperlipidemic agents use and hypertension drug use may affect the overall vascular and lipid metabolism levels, the role of AIP in the all-cause and CVD-specific mortality in patients with hypertension who had different medication situations should be further explored. Differently, the U-shape association between AIP levels and all-cause mortality was only found in females. Such gender differences in AIP values have been demonstrated in a previous fifteen-year cohort study, in which Sadeghi et al. [26] conducted on healthy adults aged over 35 years old to assess the value of AIP in the prediction of mortality. Relationship of AIP and CVD-specific mortality was also found in patients aged ≥ 60 years old. A study on predictive performance of AIP of all-cause mortality and CVD-specific mortality in middle-aged and elderly Lithuanian population showed that the risk of CVD mortality significantly increased in males with the highest AIP quintile compared with that for the lowest quintile [27]. In females, all-cause mortality was significantly higher for the 4th quintile of AIP as compared with the 1st quintile [27]. Płaczkowska et al. [28] found that different lipid parameters and AIP are associated with a high concentration of small dense LDL depending on the age group. Generally, a stronger association of small dense LDL with lipid parameters was observed in older age, which may be attributed to a longer duration of metabolic disorders. The strength and direction of these relationships observed in middle-aged and older adults differ in various studies [29–31].

This study population was from the NHANES database, which using a multistage sampling design with a weighting scheme that the sample size was large and representative. To our knowledge, it was the first time to explore the association between AIP and all-cause mortality and CVD-specific mortality in patients with hypertension. Also, we tried the best to adjust for the covariates and performed subgroup analyses to explored these associations in different populations. However, there were still some limitations in this study. The study variables including dietary and complications were collected from NHANES that were self-reported, which may lead to a recall bias. Moreover, we were unable to perform the adjustments of some other covariates due to diseases and interventions that occurred during follow-up were not available in NHANES.

## Conclusion

AIP was associated with both all-cause mortality and CVD-specific mortality in patients with hypertension in a U-shape. In clinical, the AIP level in patients with different age, gender, race, BMI, DM, CVD, antihyperlipidemic agents and hypertension drug use should be carefully monitored and controlled, in order to improve their prognosis.

## Data Availability

The datasets generated and/or analyzed during the current study are available in the NHANES database, https://wwwn.cdc.gov/nchs/nhanes/.

## List of abbreviations

CVD: Cardiovascular diseases
AIP: Atherogenic index of plasma
HDL-C: High-density lipoprotein cholesterol
RCS: Restricted cubic spline
BMI: Body mass index
NHANES: National Health and Nutrition Examination Survey
NCHS: National Center for Health Statistics
DM: Diabetes mellitus
eGFR: Estimated glomerular filtration rate
DASH: Dietary Approaches to Stop Hypertension
MET: Metabolic equivalent
PAQ: Physical activity reports
HbAlc: Glycosylated Hemoglobin
MCQ: Multiple choice question
